# Supplementary material for: Individual, Family, and Socioeconomic Contributors to Dental Caries in Children from Low- and Middle-Income Countries
Source: Int J Environ Res Public Health. 2022 Jun 10;19(12):7114. doi: 10.3390/ijerph19127114 (PMC9222700; doi:10.3390/ijerph19127114)
Supplement: Supplementary file 1 [file ijerph-19-07114-s001.zip › ijerph-1738649-supplementary.pdf]

Supplementary Tables S1-S13

Study	Total sample size	Proportion (%) with Caries	95% CI	Weight (%)
				Random
Renggli, 2021	1,307	48.125	45.385 to 50.874	9.16
Adeniyi, 2016	973	21.686	19.134 to 24.410	9.14
Ndekero, 2021	831	44.765	41.348 to 48.220	9.13
Folayan, 2019	370	4.865	2.908 to 7.580	9.03
Saraiva, 2007	3,189	28.442	26.880 to 30.042	9.19
Peres, 2009	339	51.917	46.455 to 57.346	9.02
Pérez, 2021	728	81.456	78.439 to 84.216	9.12
Fraiz, 2001	200	32.500	26.063 to 39.465	8.88
Moura-2005	343	44.315	38.981 to 49.748	9.02
Borges, 2020	1,993	41.194	39.023 to 43.392	9.18
Ayele, 2013	842	36.342	33.087 to 39.694	9.13
Total (random effects)	*11,115*	*38.696*	*28.412 to 49.518*	*100*

Table S1. Proportion of individual studies presented as percentage with 95% Confidence Intervals for Caries Prevalence in the total Sample. The table depicts the weight (%) of included studies.

Study	Sample size with Caries	Proportion (%) of Females	95% CI	Weight (%)
				Random
Renggli, 2021	629	49.762	45.784 to 53.742	31.42
Adeniyi, 2016	211	53.081	46.108 to 59.966	10.57
Ndekero, 2021	372	50.269	45.069 to 55.464	18.60
Folayan, 2019	18	61.111	35.745 to 82.701	0.95
Peres, 2009	176	43.750	36.298 to 51.416	8.83
Pérez, 2021	593	49.410	45.313 to 53.512	29.63
Total (random effects)	*1999*	*49.675*	*47.488 to 51.864*	*100*

Table S2. Proportion of individual studies presented as percentage with 95% confidence intervals for female gender prevalence in the sample with caries. The table depicts the weight (%) of included studies.

Study	Sample size	Proportion (%)	95% CI	Weight (%)
				Random
Ndekero, 2021	372	99.462	98.071 to 99.935	14.65
Folayan, 2019	18	27.778	9.695 to 53.480	12.86
Peres, 2009	176	68.750	61.343 to 75.512	14.53
Pérez, 2021	593	60.202	56.136 to 64.167	14.69
Fraiz, 2001	65	53.846	41.033 to 66.298	14.16
Moura-2005	152	54.605	46.338 to 62.689	14.49
Ayele, 2013	306	92.157	88.555 to 94.910	14.63
Total (random effects)	*1682*	*69.741*	*47.839 to 87.728*	*100.00*

Table S3. Proportion of individual studies presented as percentage with 95% confidence intervals for sugary drinks/sweets consumption in the sample with caries. The table depicts the weight (%) of included studies.

Study	Sample size	Proportion (%)	95% CI	Weight (%)
				Random
Ndekero, 2021	372	23.118	18.928 to 27.742	14.58
Folayan, 2019	18	55.556	30.757 to 78.470	12.70
Peres, 2009	176	73.864	66.719 to 80.186	14.46
Pérez, 2021	593	37.774	33.857 to 41.814	14.63
Moura-2005	152	82.237	75.218 to 87.956	14.42
Borges, 2020	821	82.339	79.555 to 84.888	14.65
Ayele, 2013	306	39.216	33.710 to 44.932	14.56
Total (random effects)	*2438*	*56.872*	*35.388 to 77.079*	*100.00*

Table S4. Proportion of individual studies presented as percentage with 95% confidence intervals for good brushing habits/ ≥2 daily teeth in the sample with caries. The table depicts the weight (%) of included studies.

Study	Sample size	Proportion (%)	95% CI	Weight (%)
				Random
Ndekero, 2021	372	14.247	10.858 to 18.218	33.17
Borges, 2020	821	3.776	2.580 to 5.317	33.97
Ayele, 2013	306	14.379	10.646 to 18.819	32.86
Total (random effects)	*1499*	*10.142*	*3.337 to 20.068*	*100.00*

Table S5. Proportion of individual studies presented as percentage with 95% confidence intervals for no educational status in the mothers in the sample with caries. The table depicts the weight (%) of included studies.

Study	Sample size	Proportion (%)	95% CI	Weight (%)
				Random
Ndekero, 2021	372	73.118	68.306 to 77.558	14.42
Saraiva, 2007	907	36.494	33.354 to 39.722	14.56
Peres, 2009	176	56.818	49.154 to 64.249	14.17
Pérez, 2021	593	54.637	50.532 to 58.697	14.51
Fraiz, 2001	65	38.462	26.654 to 51.357	13.42
Borges, 2020	821	23.264	20.414 to 26.308	14.55
Ayele, 2013	306	17.647	13.544 to 22.390	14.37
Total (random effects)	*3240*	*42.458*	*27.985 to 57.625*	*100.00*

Table S6. Proportion of individual studies presented as percentage with 95% confidence intervals for primary educational status in the mothers in the sample with caries. The table depicts the weight (%) of included studies.

Study	Sample size	Proportion (%)	95% CI	Weight (%)
				Random
Ndekero, 2021	372	12.634	9.433 to 16.445	14.49
Saraiva, 2007	907	45.865	42.585 to 49.172	14.70
Peres, 2009	176	42.045	34.659 to 49.705	14.10
Pérez, 2021	593	45.363	41.303 to 49.468	14.62
Fraiz, 2001	65	61.538	48.643 to 73.346	13.00
Borges, 2020	821	57.856	54.395 to 61.261	14.68
Ayele, 2013	306	55.556	49.794 to 61.209	14.41
Total (random effects)	*3240*	*44.958*	*33.001 to 57.218*	*100.00*

Table S7. Proportion of individual studies presented as percentage with 95% confidence intervals for secondary and/or higher educational status in the mothers in the sample with caries. The table depicts the weight (%) of included studies.

Study	Sample size	Proportion (%)	95% CI	Weight (%)
				Random
Ndekero, 2021	372	35.484	30.620 to 40.581	33.53
Moura-2005	152	84.868	78.165 to 90.160	33.02
Ayele, 2013	306	39.216	33.710 to 44.932	33.45
Total (random effects)	*830*	*54.084*	*26.632 to 80.253*	*100.00*

Table S8. Proportion of individual studies presented as percentage with 95% confidence intervals for helping child with toothbrushing in the sample with caries. The table depicts the weight (%) of included studies.

Study	Sample size	Proportion (%)	95% CI	Weight (%)
				Random
Ndekero, 2021	372	52.151	46.940 to 57.326	49.71
Saraiva, 2007	907	26.902	24.040 to 29.915	50.29
Total (random effects)	1279	39.095	16.584 to 64.403	100.00

Table S9. Proportion of individual studies presented as percentage with 95% confidence intervals for children with caries using fluoride toothpaste. The table depicts the weight (%) of included studies.

Study	Sample size	Proportion (%)	95% CI	Weight (%)
				Random
Renggli, 2021	629	40.223	36.365 to 44.173	14.58
Folayan, 2019	18	44.444	21.530 to 69.243	12.72
Saraiva, 2007	907	10.254	8.356 to 12.414	14.60
Peres, 2009	176	22.727	16.761 to 29.636	14.41
Pérez, 2021	593	52.614	48.506 to 56.695	14.58
Borges, 2020	821	10.231	8.243 to 12.510	14.60
Ayele, 2013	306	81.699	76.903 to 85.870	14.51
Total (random effects)	3450	35.904	16.732 to 57.788	100.00

Table S10. Proportion of individual studies presented as percentage with 95% confidence intervals to determine low socioeconomic status of children/their families with caries. The table depicts the weight (%) of included studies.

Study	Sample size	Proportion (%)	95% CI	Weight (%)
				Random
Renggli, 2021	629	27.186	23.744 to 30.844	14.65
Folayan, 2019	18	22.222	6.409 to 47.637	12.39
Saraiva, 2007	907	22.161	19.497 to 25.007	14.67
Peres, 2009	176	49.432	41.826 to 57.057	14.44
Pérez, 2021	593	30.691	26.999 to 34.577	14.64
Borges, 2020	821	77.345	74.325 to 80.167	14.66
Ayele, 2013	306	18.954	14.719 to 23.802	14.56
Total (random effects)	3450	35.388	18.044 to 55.001	100.00

Table S11. Proportion of individual studies presented as percentage with 95% confidence intervals to determine middle socioeconomic status of children/their families with caries. The table depicts the weight (%) of included studies.

Study	Sample size	Proportion (%)	95% CI	Weight (%)
				Random
Renggli, 2021	629	18.283	15.338 to 21.530	22.61
Folayan, 2019	18	33.333	13.343 to 59.007	11.11
Saraiva, 2007	907	34.620	31.523 to 37.817	22.83
Peres, 2009	176	25.000	18.790 to 32.072	20.89
Pérez, 2021	593	16.695	13.781 to 19.945	22.56
Total (random effects)	2323	24.511	16.212 to 33.898	100.00

Table S12. Proportion of individual studies presented as percentage with 95% confidence intervals to determine high socioeconomic status of children/their families with caries. The table depicts the weight (%) of included studies.

Study	Sample size	Proportion (%)	95% CI	Weight (%)
				Random
Adeniyi, 2016	973	15.930	13.685 to 18.383	20.03
Saraiva, 2007	1596	56.454	53.980 to 58.904	20.08
Pérez, 2021	728	45.879	42.214 to 49.578	19.99
Peres, 2009	339	46.313	40.910 to 51.781	19.80
Borges, 2020	1993	60.763	58.579 to 62.914	20.10
Total (random effects)	5629	44.438	27.728 to 61.824	100.00

Table S13. Proportion of individual studies presented as percentage with 95% confidence intervals to determine the access to dental services among both groups. The table depicts the weight (%) of included studies.
